# Antimicrobial and antibiofilm actions of iridoids: a comprehensive mechanistic review

**DOI:** 10.1007/s00210-026-05063-9

**Published:** 2026-03-02

**Authors:** Ahmed H. Elosaily, Ahmed M. El-Dessouki, Marwa Ali-Tammam, Mona Ismail, Momen Lotfy, Haidy A. Abbas, Mohamed S. Abd El Hafeez, Riham A. El-Shiekh, Dalia El Amir, Ghadir A. Sayed

**Affiliations:** 1 Department of Pharmacognosy, Faculty of Pharmacy, Ahram Canadian University, Giza, 12573 Egypt; 2 Pharmacology and Toxicology Department, Faculty of Pharmacy, Ahram Canadian University, 6th of October City, Giza, 12566 Egypt; 3 Department of Microbiology & Immunology, Faculty of Pharmacy, Future University in Egypt, Cairo, 12311 Egypt; 4 Department of Pharmacognosy, Faculty of Pharmacy, Beni-Suef University, Beni-Suef, 62514 Egypt; 5 Department of Pharmacy, Kut University College, Al Kut, Wasit, 52001 Iraq; 6 Department of Pharmacognosy, Faculty of Pharmacy, Egyptian Russian University, Cairo-Suez Road, Badr City, 11829 Egypt; 7 Department of Pharmacognosy, Faculty of Pharmacy, Cairo University, Kasr El-Aini Street, Cairo, 11562 Egypt; 8 Department of Biochemistry, Faculty of Pharmacy, Egyptian Russian University, Badr City, Cairo, 11829 Egypt

**Keywords:** Bioactive, Natural sources, Antimicrobial, Herbal molecules, Drug resistance

## Abstract

The intensive use of antibiotics resulted in the development of resistance, posing a significant global threat to human health. Natural products have garnered significant attention in modern drug discovery and development. Utilizing natural compounds and extracts, either alone or in combination with conventional antimicrobial drugs, could be a significant development in overcoming pathogen resistance mechanisms. Among these natural compounds, iridoids exhibited diverse biological activities, such as anti-inflammatory, antioxidant, anticancer, antidiabetic, antimicrobial, and neuroprotective properties. They are present in some widely available fruits, such as olives, noni, and berries. In this concise review, the utilization of iridoids as a promising approach to combat antimicrobial resistance is discussed. Iridoids exert their antimicrobial effects through various mechanisms including inhibition of microbial growth, disruption and permeability of microbial cell membranes, interference with metabolic pathways, and inhibition of bacterial efflux pumps. Their structural versatilities give them an advantage in targeting a variety of microorganisms.

**Graphical Abstract:**

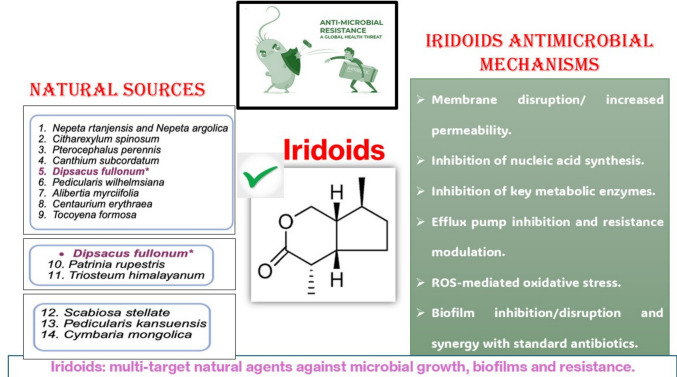

**Supplementary Information:**

The online version contains supplementary material available at 10.1007/s00210-026-05063-9.

## Introduction

Natural products have garnered significant attention in modern drug discovery and development (Sobhy et al. 2024; Allam et al. 2025; Azzam et al. 2025). Iridoids are found in large amounts in many plants, such as members of the Rubiaceae, Apocynaceae, and Plantaginaceae families (Thu et al. 2022). They are also present in medicinal plants traditionally used for centuries, including valerian, olive, and gardenia, leading to much interest among researchers in unraveling their biological activities. Iridoids demonstrate several therapeutic potentials, including anti-inflammatory, antioxidant, and antimicrobial effects (Mori et al. 2021; Weng et al. 2021). Due to the rising global concern of antibiotic resistance, iridoids emerge as promising natural alternatives (Hanh et al. 2016; El-Shiekh et al. 2024). Iridoids interfere with the normal physiological process of various microbes in multiple ways, such as damaging the cell wall or membrane, impeding essential enzymes, or interfering with replication processes in pathogens (Joubouhi et al. 2017a; Liu et al. 2017). Biofilm formation, especially on indwelling medical devices such as catheters, could result in infections and substantially affect patients’ quality of life (Dousari et al. 2024). The effect of iridoids on biofilms has expanded their potential applications, especially in the management of chronic infections where biofilm formation contributes significantly to the infection process (Priyanto et al. 2022). For instance, iridoids present in *Gardenia jasminoides* are active against biofilm-forming bacteria such as *Pseudomonas aeruginosa*, which is a well-known causative agent of hospital-acquired infections. Iridoids, acting as a double-headed arrow targeting both the microbes and biofilm matrices, might offer a significant improvement in the effectiveness of antimicrobial treatment (Al Kateeb et al. 2022; Alfeqy et al. 2024). The search for such naturally occurring products with a broader spectrum of antimicrobial effects is now on the rise (Costa et al. 2023). This review will thoroughly investigate what has been known thus far about iridoids, especially in light of their potential as antimicrobial and antibiofilm agents.

## Search strategy

A narrative review with extensive data from various databases such as the Egyptian Knowledge Bank (EKB), Scopus, Web of Science, PubMed, Google Scholar, and Elsevier databases was gathered until 2024. All keywords pertaining to “iridoids,” “secoiridoids,” “antimicrobial,” “antibiofilm,” “MIC,” “nomenclature,” “phytochemical classification,” “isolation,” “structures,” “applications,” “in vitro,” OR “in vivo,” “biological activities,” “mechanisms of actions,” and “plant extracts enriched in iridoids” were utilized in the search, including results from peer-reviewed English-language research and review articles.

## Nomenclature of iridoids

Iridoids are highly oxygenated monoterpenes represented by the cyclopenta[c] pyranoid skeletal, which is based on the structure of monoterpene iridane (Sobhy et al. 2024). These compounds are well spread as secondary plant metabolites in the angiosperm plant families and some ant species. Often, these iridoids in several plants are 7,8-secoderivatives called secoiridoids because these are produced due to the scission of the cyclopentane ring at the C-7–C-8 bond. These compounds can be found in around 57 plant families of dicotyledons, mostly in the Asterids clade regarding the great structural diversities (Dinda et al. 2011; Kou et al. 2022; Grover et al. 2023). Although these compounds were first isolated from plants in the 1800 s, the very initial works that were done on the structures of this class of compounds commenced only after the isolation of iridodial, iridomyrmecin, and isoiridomyrmecin (= iridolactone) from some species of Iridomyrmex, a genus of ants mostly found in Australia (namely *I. detectus*, *I. humilis*, *I. conifer*, *I. nitidus*, and *I. purpureus*) (Jensen et al. 2002).

## Phytochemical classification of iridoids

Iridoids are a significant class of phytochemicals with widely recognized biological features (Ludwiczuk et al. 2017) that include a wide group of cyclopentane pyran monoterpenoids. They are distinguished by skeletons in which a six-membered ring containing an oxygen atom is fused to a cyclopentane ring. These molecules most usually occur in plants paired with sugars; therefore, they are categorized as glycosides, which are further separated into four main classes: iridoid glycosides, seco-iridoid glycosides, simple iridoid, and bis-iridoids (Wang et al. 2020). Orobanchaceae, Lamiaceae, and Valerianaceae are the main families in which iridoids are identified or isolated. The iridoid glycosides can be divided into five subcategories: iridoid glycosides having an eight-carbon basic skeleton; iridoid glycosides with a nine-carbon basic skeleton; iridoid glycosides with a ten-carbon basic skeleton; iridoid glycosides with a fourteen-carbon basic skeleton or iridoid glycosides of plumeria type; and alkaloid-conjugated iridoid glycosides (Dinda and Dinda 2019). This last category includes two subtypes: simple ten-carbon iridoid glycosides and iridoid glycosides related to valeriana type. Iridoid aglycones fall under the categories of four groups: simple iridoid aglycone and their derivatives, rearranged iridoid aglycones, iridoid aglycones of valeriana type, and iridoid aglycones of plumeria type. In addition, these secoiridoid glycosides are classified into four subgroups: simple secoiridoid glycosides, terpene-conjugated secoiridoid glycosides, phenolic-conjugated secoiridoid glycosides, and alkaloid-conjugated secoiridoid glycosides (Dinda and Dinda 2019).

1. Iridoids glycosides

Iridoid glycosides are monoterpenoid phytoconstituents naturally occurring in the plant kingdom, particularly within families like Plantaginaceae, Scrophulariaceae, Lamiaceae, Apocynaceae, and Gentianaceae (Rahamouz-Haghighi 2023), which is characterized by a cyclopentanopyran ring structure, generally linked to sugar moieties, forming glycosides. The basic iridoid structure consists of a six-membered ring fused to a five-membered ring (Viljoen et al. 2012). Many biological activities have been proved for several members of iridoid glycosides, such as the anti-inflammatory (Carrillo-Ocampo et al. 2013), hepatoprotective (Bridi et al. 2023), and neuroprotective (Rahamouz-Haghighi 2023) properties of aucubin and catalpol iridoids. Others were known for their roles in metabolic regulation, such as loganin (Xu et al. 2022). Other members of iridoid glycosides were mentioned in Table S1.

2. Secoiridoid glycosides

Secoiridoids are a subclass in which the cyclopentane ring is cleaved, resulting in an open-ring structure. This cleavage often enhances their bioactivity, particularly in terms of antioxidant and anti-inflammatory properties. Secoiridoids commonly exhibit one or more hydroxyl groups and can also exist in glycosylated forms (Huang et al. 2019). Prominent examples of secoiridoids include oleuropein, predominantly found in olive leaves and oil, renowned for its antioxidant, anti-inflammatory, and cardioprotective benefits (Hassen et al. 2015). Another notable secoiridoid is swertiamarin, derived from Swertia species, which is currently being studied for its potential anti-diabetic and hepatoprotective effects besides its protective role in cardiac and metabolic diseases (Leong et al. 2016). Other members of secoiridoids’ glycosides were stated in Table S1.

3. Non-glycosidic iridoids

Non-glycosidic iridoids are free from sugar moieties, resulting in increased lipophilicity and potential for rapid absorption. This class is less common than glycosidic iridoids but is important due to its high bioavailability.

Valepotriates, found in *Valeriana officinalis* (Valerian root), are unique compounds known for their calming and muscle-relaxing effects; they are the famous example of non-glycosidic iridoids. Dihydrovaltrate, another compound from valerian, works in a similar way, making it a reliable option for managing stress and sleep issues (Solati and Motlagh 2008). Aucubigenin, which comes from the breakdown of aucubin in *Plantago major*, is valued for its strong anti-inflammatory and antioxidant benefits, often used in herbal remedies (Kartini et al. 2023). Other examples are mentioned in TableS1.

4. Bis-iridoids

A specific type of dimeric iridoids known as bis-iridoids is created when two iridoid units structurally combine to produce one (Li et al. 2013). Cantleyoside, laciniatosides I–II, sylvestrosides I–III, and their dimethyl acetals are notable examples; these compounds were mostly isolated from Pterocephalus hookeri (C.B. Clarke) Höeck. These substances have been shown to have strong analgesic and anti-inflammatory properties (Chen et al. 2018). The characteristic coupling between the C-7 location of the iridoid and the C-4 position of the secoiridoid distinguishes sclerochitonoside C, another bis-iridoid. This structure includes subunits derived from the secoiridoid analogue of 8-epiloganic acid and its 4′-hydroxyphenylethyl esters (Scott Brown et al. 2011). Table S1 also includes other examples of bis-iridoids.

## Antimicrobial and antibiofilm activity of plant extracts enriched in iridoids

**Fig. 1:**
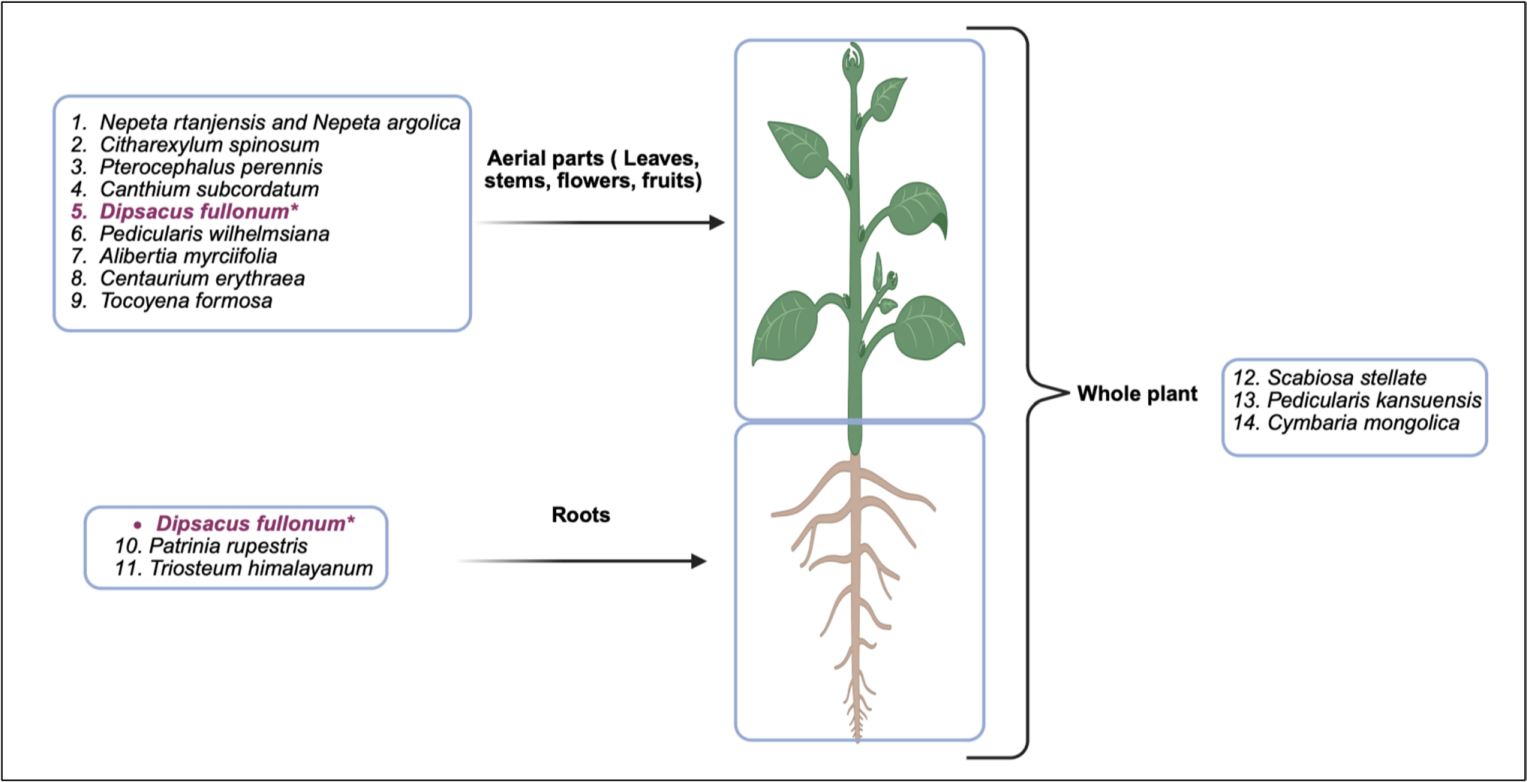
Schematic representation of plant parts identified as sources of isolated iridoids exhibiting antimicrobial activity

Plant extracts rich in iridoids show promising antimicrobial and antibiofilm activity against various pathogens as shown in Fig. 1 and Table 1.

**Table 1:** Antimicrobial activities of plant extracts and isolated iridoid compounds obtained from different plant parts compared to benchmark antimicrobials (listed in the same order as presented in Fig. 1)

#	Plant source	Extract/compound	Target organisms	Main effect	Standard antibiotic/antifungal	Reference(s)
1	*Nepeta* spp.	Isolated iridoids (*cis*, *trans*-NL, *trans*, *cis*-NL)	*Listeria monocytogenes, Salmonella typhimurium, Penicillium ochlochloron*	Significantly higher activity. 80-fold and 20-fold more potent than streptomycin (bacteria); 200-fold more potent than ketoconazole (fungus)	Streptomycin; ketoconazole	Aničić et al. (2021)
2	*Citharexylum spinosum*	Methanol bark extract	*Staphylococcus aureus*	High activity. Maximum inhibition zone of 44.5 mm	Not determined	Ajaib et al. (2017); Abd-Alla et al. (2024)
3	*Pterocephalus perennis*	Methanol extract and isolated iridoids	6 bacterial strains; 3 *Candida* species	Lower activity. Showed only moderate inhibition zones (8–12 mm) compared to large zones from standard antibiotics (20–25 mm)	Amoxicillin/clavulanic, netilmicin	Graikou et al. (2002)
4	*Canthium subcordatum*	Isolated iridoids (e.g., canthiumoside)	*Vibrio cholerae, Shigella flexneri, S. aureus*	Lower activity. MIC values 2–4 times higher than ampicillin/ciprofloxacin; overall potency critically limited	Ampicillin, ciprofloxacin	Joubouhi et al. (2017b)
5	*Dipsacus fullonum*	Water extracts of leaves and roots	*E. coli, S. aureus*	Significant activity against specific strains, but no effect on others	Not compared to mentioned standard	Oszmiański et al. (2020)
6	*Pedicularis wilhelmsiana*	40–60% methanol/water fractions	*Pseudomonas aeruginosa, S. epidermidis, S. aureus*	Higher activity than the crude methanolic extract	Not determined	Khodaie et al. (2019)
7	*Alibertia myrciifolia*	Geniposidic acid	*Colletotrichum gloeosporioides*	Lower activity. Inhibited mycelial growth at 150 μg/mL, while standard was effective at 2 μg	Nistatin	Luciano et al. (2010)
8	*Centaurium erythraea*	Gentiopicroside and crude extract	12 of 17 pathogenic bacterial species (e.g., *Serratia marcescens*)	Significantly lower activity. Extract was 10^3^- to 10^6^-fold less potent than ciprofloxacin	Ciprofloxacin	Kumarasamy et al. (2003a)
9	*Tocoyena formosa*	Total extract and isolated epimeric mixture (α and β-gardiol)	DNA-repair-deficient and wild-type *Saccharomyces cerevisiae*	IC12 values provided	Not determined	Bolzani et al. (1997)
10	*Patrinia rupestris*	Isolated iridoids (rupesin A–E, etc.)	*Bacillus subtilis, E. coli, S. aureus*	Lower activity. Showed weak to moderate inhibition zones (10–13 mm) compared to chloramphenicol (16–20 mm)	Chloramphenicol	Yang et al. (2006)
11	*Triosteum himalayanum*	Total extract and isolated iridoid (triohima B)	*Peronophythora litchi*	Significant activity reported (85% inhibition, EC50 34.0 μg/mL),	Not determined	Li et al. (2009)
12	*Scabiosa stellata*	Ethanol extract and isolated iridoids	17 bacterial strains (strongest vs. *Streptococcus pyogenes*)	Lower activity. Extract was markedly less potent than standard agents (gentamicin, vancomycin)	Gentamicin, vancomycin	Lehbili et al. (2018)
13	*Pedicularis kansuensis*	Four isolated iridoids (e.g., ixoroside, aucubin)	*E. coli, S. aureus*	Strong activity reported with inhibition zones of 13–20 mm	Not determined	Yuan et al. (2003)
14	*Cymbaria mongolica*	Non-glycosidic iridoids (e.g., compound 1)	*B. subtilis, E. coli, S. aureus*	Comparable activity. Exhibited antibacterial activity close to that of chloramphenicol	Chloramphenicol	Dai et al. (2002)

1. *Nepeta rtanjensis* and *N. argolica*

In vitro antimicrobial activity of the methanolic leaf extract of both *Nepeta* species (*N. rtanjensis* and *N. argolica*) (family *Lamiaceae*) along with the isolated iridoid (*trans,cis*-nepetalactone (NL), *cis,trans*-nepetalactone (NL), and 1,5,9-epideoxyloganic acid (eDLA)) were evaluated against eight different bacterial strains (*Bacillus cereus*, *Staphylococcus aureus*, *Micrococcus flavus*, *Listeria monocytogenes*, *Enterococcus faecalis*, *Pseudomonas aeruginosa*, *Escherichia coli*, and *Salmonella typhimurium*). As well as seven food-borne fungal pathogens (*Aspergillus ochraceus*, *A. fumigatus*, *A. versicolor*, *A. niger*, *A. funiculosum*, *P. ochlochloron*, and *P. v. cylopium*). Benchmarking against streptomycin revealed that the activity of the purified iridoids was not only significant but often substantially exceeded that of the relevant used antibiotic. In most tests, the MIC values for the isolated iridoids (cis,trans-NL, trans, cis-NL, and 1,5,9-eDLA) are lower than those for streptomycin, indicating greater antibacterial potency. Isolated iridoid *cis,trans*-nepetalactone demonstrated remarkable potency, with an MIC of 0.0025 mg/mL against *Listeria monocytogenes* and *Salmonella typhimurium*, making it 80-fold more potent than the standard antibiotic streptomycin, Moreover, *trans,cis*-nepetalactone (*trans,cis*-NL) with MIC of 0.001 mg/ml against *Listeria monocytogenes* was 20-fold more potent that streptomycin as well. Similarly, the antifungal activity of the iridoids; cis,trans-nepetalactone and *trans,cis-nepetalactone* (MIC, 0.005 mg/mL) was 200-fold more potent than the standard antifungal ketoconazole against *Penicillium ochlochloron*, while the crude leaf extracts themselves showed strong activity (MICs from 0.05 to 0.4 mg/mL). This direct comparison confirms that the isolated iridoids are the principal active constituents, displaying MIC values indicative of strong, clinically relevant potency against a broad spectrum of pathogens (Aničić et al. 2021). Notably, purification is essential, as the crude extracts exhibited lower activity than the isolated compounds, confirming that the iridoids are the primary bioactive constituents.

2 *Citharexylum spinosum*

The *Citharexylum* genus is a valuable source of iridoid glycosides. All the iridoids extracted from *Citharexylum* species possess a mono-glucosidic structure. Various parts of *Citharexylum spinosum* (Verbenaceae family) were tested to evaluate their antimicrobial effect using the agar well diffusion method. The methanol bark extract showed the maximum antibacterial effect against *Staphylococcus aureus* with an inhibition zone of 44.5 ± 0.5 mm, while the chloroform extract of leaves showed minimum activity with an inhibition zone of 10.5 ± 0.5 mm (Ajaib et al. 2017; Abd-Alla et al. 2024).

3 *Pterocephalus perennis*

In vitro antimicrobial activity of the methanol extract of the aerial parts of *P. perennis* (Caprifoliaceae family) along with their isolated iridoids (loganin, loganic acid, cantleyoside, secologanin, secologanin-dimethyl-acetal, Cantleyoside-dimethyl-acetal) was tested against six bacterial strains (*Staph. aureus*, *Staph. epidermidis*, *P. aeruginosa*, *E. coli*, *Enterobacter cloacae*, *K. pneumoniae)* in addition to three pathogenic fungi (*Candida albicans*, *C. tropicalis*, and *C. glabrata*) using the disk diffusion method. The antimicrobial efficacy of the crude extract and compounds 1–6 showed lower activity compared to that of the standard antibiotics: amoxicillin with clavulanic acid and netilmicin. The standards consistently produced large zones of inhibition (20–25 mm) across both Gram-positive and Gram-negative bacteria. In contrast, the tested compounds and extract exhibited only moderate activity, with inhibition zones in the (8–12 mm) range. Only the compounds 1 and 6 and the extract showed moderate activity against Candida species with an inhibition zone of (9–12 mm). The antifungal agent amphotericin B produced a larger inhibition zone of 23–25 mm, indicating lower activity of the isolated iridoids and the extract compared to the standard (Graikou et al. 2002). These results indicate that, although the methanol extract and its iridoid constituents possess measurable antimicrobial properties, their overall activity is limited relative to clinically used antibiotics and antifungal agents.

4 *Canthium subcordatum*

In the study conducted by Joubouhi et al. (2017b), in vitro antibacterial activity of *Canthium subcordatum* fruits (Rubiaceae family) was tested for the methanol extract, hexane, ethyl acetate, and iso-butanol fractions beside the isolated iridoid compounds (canthiumoside (1–5), shanzhigenin methyl ester (6), linearin (7), mussaenoside (8), and shanzhiside methyl ester (9)) against *Vibrio cholerae, Shigella flexneri*, and *Staph. aureus* using the broth micro-dilution method. *C. subcordatum* fruits showed different degrees of antibacterial activity with MIC range between 128 and 512 ug/mL, while iridoids; canthiumoside 1 (1), and linearin (7) were the most active antibacterial compounds with MIC values of 8–64 µg/mL, yet it remained 2–4 times less potent than the standard antibiotics ampicillin and ciprofloxacin against susceptible strains. Moreover, based on the MIC/MBC ratio, the isolated iridoids (compounds 5–9) demonstrated significantly weaker antibacterial activity compared to the standard antibiotics. While most iridoids exhibited a bactericidal effect (MBC/MIC ratios of 1–2), their MIC values ranged from 32 to 256 µg/mL, substantially higher than ciprofloxacin (2–16 µg/mL) against tested strains. These results confirm that while the iridoids possess bactericidal properties, their overall potency and spectrum are critically limited compared to conventional antibiotics (Joubouhi et al. 2017b).

5 *Dipsacus fullonum*

In the leaves and roots of *Dipsacus fullonum* L. (Caprifoliaceae family), five iridoid compounds were detected (loganic acid, loganin, sweroside, cantleyoside, and sylvestroside III). The water extracts of the leaves and roots of *D. fullonum* were tested against five bacterial strains: *B. subtilis*, *E. coli*, *P. aeruginosa*, *P. fluorescens*, and *S. aureus*. Additionally, the activity of the extract was analyzed against selected yeasts: *Candida famata*, *Candida tropicalis*, *Candida sphaerica*, *Saccharomyces cerevisiae*, and *Yarrowia lipolytica*. The extracts showed significant antibacterial effects against *E. coli* and *S. aureus*. However, no other positive or negative effects of the plant materials were observed in the other tested bacteria and yeasts. The standard antimicrobial agents used in the study were oxytetracycline (5 µg/mL) (for bacteria) and cycloheximide (20 mg/mL) (for yeasts) (Oszmiański et al. 2020).

6 *Pedicularis wilhelmsiana*

In vitro antibacterial effects of methanol/water fractions obtained from *Pedicularis wilhelmsiana* (Scrophulariaceae family) were tested against *P. aeruginosa*, *S. epidermidis*, *S. aureus*, and *Micrococcus luteus* using the agar well diffusion method. The results showed that the antimicrobial effect of 40% and 60% methanol/water fractions was more active than the methanolic extract. Moreover, three iridoids—Aucubin, ipolamiid, and 5-hydroxy-8-epi-loganin—were isolated from the aerial parts of *Pedicularis wilhelmsiana* (Khodaie et al. 2019)*.*

7 *Alibertia myrciifolia*

The antifungal activities of the iridoids (10-*O*-vanilloyl-geniposidic acid and geniposidic acid) isolated from the butanol fraction of the aerial parts of *Alibertia myrciifolia* (Rubiaceae family) were evaluated against three fungal strains: *Colletotrichum gloeosporioides*, *Fusarium solani*, and *Aspergillus niger* using agar cup plate assay. Nistatin (2 μg) was used as a positive control. The result showed that geniposidic acid inhibited the development of the mycelial mass of *C. gloeosporioides* at a concentration of 150 µg/mL, while 10-*O*-vanilloyl-geniposidic acid showed no inhibition at a concentration of 112.5 μg/mL (Luciano et al. 2010).

8 *Centaurium erythraea*

Gentiopicroside (1), a secoiridoid glycoside isolated from the methanol extract of the aerial parts of *Centaurium erythraea* (Gentianaceae family), was tested against 17 bacterial species including *B. subtilis*, *B. cereus*, *Citrobacter freundii*, *E. coli*, *Proteus mirabilis*, *S. aureus*, *S. epidermidis*, and *Serratia marcescens*. The results showed that Gentiopicroside inhibited the growth of 12 of the 17 pathogenic bacterial species tested. Moreover, when compared with ciprofloxacin, the crude extract exhibited markedly lower antibacterial potency based on the MIC values. The extract was between 10^4^- and 10^6^-fold less potent than ciprofloxacin against most Gram-positive and Gram-negative bacteria. While the strongest relative activity was observed against *Serratia marcescens* (approximately 2.5 × 10^3^-fold weaker) (Kumarasamy et al. 2003a).

9 *Tocoyena formosa*

The antifungal activity of the total leaves extract of *Tocoyena formosa* (Rubiaceae family) along with the isolated epimeric mixture of *α* and *β-*gardiol was tested against DNA repair-deficient yeast strains and the wild type strain of *Saccharomyces cerevisiae*. The total extract showed fungicidal activity with an IC_12_ of 980, 950, 650, and 680 µg/mL, respectively, while the isolated *α* and *β-*gardiol showed activity with an IC_12_ of 120, 126, 90, and 105 µg/mL, respectively (Bolzani et al. 1997).

10 *Patrinia rupestris*

Antibacterial activities of 11 isolated iridoids ((Rupesin A–E (1–5), Sarracenin (6), Patriscabrol (7), Villosol (8), 3-patriscabrol (9), Patriscabroside II (10), and 7-ketologanin (11)) from the ethyl acetate and butanol fractions of the roots of *Patrinia rupestris* (Caprifoliaceae family) were tested against *B.*, *E. coli*, and *S. aureus*, using cup-plate method with chloramphenicol as a positive control. The antibacterial evaluation of compounds 1–11 showed markedly lower potency compared to chloramphenicol. While chloramphenicol consistently showed strong activity (16–20 mm inhibition zone) across all species, most tested compounds exhibited weak to moderate activity (10–13 mm inhibition zone). Only compounds 4, 7, 9, 10, and 11 showed enhanced activity, particularly against *S. aureus* and *E. coli*, yet remained below the activity of the reference antibiotic (Yang et al. 2006).

11 *Triosteum himalayanum*

The antifungal activity of the total extract of *Triosteum himalayanum* (Caprifoliaceae family) and the isolated iridoid compound (Triohima B) was tested against the plant pathogenic fungus *Peronophythora litchi*. The results showed that the total extract had significant inhibitory activity against *P. litchi* with 85% inhibition at 5 mg/mL, while Triohima B showed inhibitory activity with an EC_50_ value of 34.0 µg/mL (Li et al. 2009).

12 *Scabiosa stellate*

Antimicrobial screening of the ethanol extract obtained from the whole plant of *S. stellata* (Caprifoliaceae family) and two isolated iridoids (7-*O*-caffeoyl-sylvestroside I and 7-*O*-(*p*-coumaroyl)-sylvestroside I) was performed against 17 bacterial strains. The ethanol extract exhibited the strongest antimicrobial activity against *Streptococcus pyogenes* (MIC = 1.2 mg/mL), while moderate-to-low activity was shown against *S. epidermidis* (MIC = 2.5 mg/mL) and *B. subtilis* (MIC = 10 mg/mL). When benchmarked against standard antimicrobial agents: gentamicin, vancomycin, and amphotericin B, the ethanol extract was markedly less potent. The isolated compounds exhibited significant antibacterial activity with MIC values against both *Enterococcus faecalis* and *S. epidermidis* and *S. aureus* with MIC values of 31.2 μg/mL and 62.5 μg/mL, respectively (Lehbili et al. 2018).

13 *Pedicularis kansuensis*

Antibacterial activities of ten iridoids (Kansuenoside, Kansuenin, Ixoroside, Euphroside, Mussaenoside, Boschnaloside, 7-deoxy-8-epi-loganic acid, 8-*epi*-loganic acid, Aucubin, and Geniposidic acid) isolated from the butanol fraction of the whole plant of *Pedicularis kansuensis* (Scrophulariaceae family), were tested against *B. subtilis*, *E. coli*, and *S. aureus*. Four compounds (Ixoroside, Boschnaloside, 8-*epi*-loganic acid, and Aucubin) showed strong antibacterial activity against *E. coli* and *S. aureus* with inhibition zones of 13 and 20 mm in diameter, respectively (Yuan et al. 2003).

14 *Cymbaria mongolica*

Antibacterial activities of 11 non-glycosidic iridoids isolated from the acetone extract of the whole plant of *Cymbaria mongolica* (Scrophulariaceae family) were assayed against *B. subtilis*, *E.coli*, and *S.aureus* and compared with that of chloramphenicol as a standard. The result demonstrated that these non-glycosidic iridoids, particularly compound 1, exhibited antibacterial activity against *B. subtilis*, *E. coli*, and *S. aureus* with inhibition zone of 14.5, 13.9, and 14.5 mm in diameter, respectively, reflecting antibacterial activity close to that of chloramphenicol in the study results (Dai et al. 2002).

## Antimicrobial and antibiofilm activity of the isolated iridoids

**Fig. 2:**
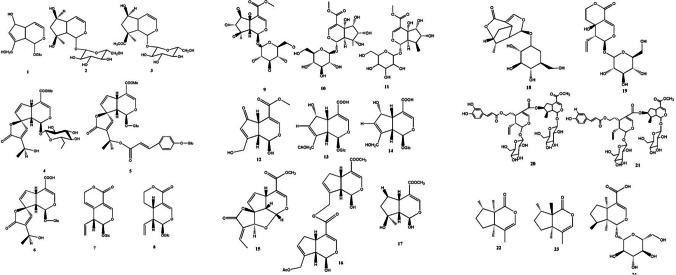
Structures of iridoids with reported antimicrobial activity

Iridoids made up of numerous unique bicyclic monoterpenes which are distributed widely in plants and fungi (Dinda et al. 2009). They constitute a large and still expanding class of cyclopentane pyran monoterpenes. They are composed of two basic carbon frameworks, substituted iridoids, and secoiridoids (Wang et al. 2018). Many plant species include bioactive iridoids as their main ingredient. Iridoids enclose a broad spectrum of pharmacological properties, such as anticancer, anti-inflammatory, antifungal, and antibacterial activities (Geu-Flores et al. 2012). They are more prevalent in the kingdom Plantae, mainly in the dicotyledonous plants from Scrophulariaceae, Pyrolaceae, Oleaceae, Labiatae, Rubiaceae, and Gentianaceae. Herein, we reviewed briefly some reported iridoids that possess antimicrobial and antibiofilm activities as listed in Table 2 and Fig. 2 .

**Table 2:** List of isolated iridoids and their antimicrobial activities

#	Iridoid/compound	Target organisms	Main effect	Reference(s)
1	Aucubin **(1)**	*S. aureus, E. coli*	Aglycone aucubigenin active against *S. aureus*	Davini et al. (1986); Lee et al. (1986)
2	Ajugol **(2)** Ajugoside **(3)**	*S. aureus, E. coli, P. aeruginosa, C. albicans, C. neoformans*	Higher activity vs Gram-positive *S. aureus*; ajugoside moderate activity	Ezer et al. (1995); Akcoş et al. (1998)
3	Plumieride **(4)** Protoplumericin A **(5)** Plumieride acid **(6)**	Various bacteria & fungi	Distinct antibacterial and antifungal activity	Afifi et al. (2006)
4	Gentiopicroside **(7)**	*Serratia marcescens*	Significant antibacterial activity (MIC 6.3 × 10⁻^3^ mg/mL)	Kumarasamy et al. (2003b)
5	Sweroside (**8)**	*B. cereus, B. pumulus, B. subtilis, Micrococcus kristinae, S. aureus, E. coli, K. pneumoniae, P. aeruginosa, Enterobacter cloacae*	Moderate antibacterial activity	Horn et al. (2001)
6	Phloyoside I **(9)**	*B. cereus, C. freundii, P. mirabilis, P. aeruginosa, S. aureus*	Low to moderate antibacterial activity (MIC 0.05–0.50 mg/mL)	Modaressi et al. (2009)
7	Phlomiol **(10)**		Low to moderate antibacterial activity	
8	Pulchelloside I **(11)**	*B. cereus*, penicillin-resistant *E. coli, P. mirabilis, S. aureus*	Significant antibacterial activity (MIC 0.05 mg/mL vs some strains)	
9	6-oxo-genipin **(12)**	*S. aureus, A. calcoaceticus, S. plymuthica, P. stutzeri, E. coli*	Significant inhibitory efficacy, especially against *S. aureus*	Kouam et al. (2013)
10	Asperulosidic acid **(13)** Deacetylasperulosidic acid **(14)**	*S. aureus* subsp. *aureus*	Moderate antibacterial activity (MIC ≈100 μg/mL)	Hanh et al. (2016)
11	Plumericin **(15)**	*E. faecalis, B. subtilis*	Strong antibacterial activity; MICs better than cloxacillin	Saengsai et al. (2015)
12	Canthiumoside **(16)** Linearin **(17)**	*Shigella flexneri, Vibrio cholerae, Staphylococcus aureus*	Antibacterial activity (MIC 8–64 μg/mL); *S. aureus* most sensitive	Joubouhi et al. (2017b)
13	Physaloside A **(18)**	*Yersinia enterocolitica, B. cereus, E. coli, S. aureus*	Comparable inhibition; at 60–90 ppm stronger vs *S. aureus* than zerumbone	Wang et al. (2018)
14	Swertiamarin **(19)**		Similar MIC vs *B. cereus*; higher MICs vs other bacteria	
15	7-O-caffeoyl-sylvestroside I **(20)** 7-O-(p-coumaroyl)-sylvestroside I **(21)**	*Enterococcus faecalis, S.epidermidis, other Gram* ±	Strong antibacterial activity; MIC 31.2 μg/mL *(E. faecalis),* 62.5 μg/mL *(S. epidermidis)*	Lehbili et al. (2018)
16	cis,trans-nepetalactone **(22)** trans,cis-nepetalactone **(23)** 1,5,9-epideoxyloganic acid **(24)**	*Listeria monocytogenes, B. cereus, S. aureus, Enterococcus faecalis, P. aeruginosa, E. coli,* multiple fungi	Significant antibacterial, antifungal and antibiofilm activities. Highly active antibiofilm activity	Aničić et al. (2021)

Aucubin (**1**), an iridoid glycoside, exhibited antimicrobial activity against Gram-positive *S. aureus* that appeared to be more sensitive to aucubin’s aglycone, aucubigenin, than Gram-negative *E. coli*. (Lee et al. 1986).

Moreover, on a study on iridoid glycoside ajugol and ajugoside isolated from a *Sideritis lycia* Boiss. and Heldr, it was demonstrated that ajugol possesses higher antibacterial activity against *S. aureus* ATCC 25923 compared to its action on *E. coli* ATCC 25882, *P. aeruginosa* RSKK 356 and yeast-like fungi *Cryptococcus neoformans* and *C. albicans* ATCC 64550 (Ezer et al. 1995; Akcoş et al. 1998). In another study, the aglycone aucubigenin, produced by the hydrolysis of aucubin, showed a strong inhibitory effect on the growth of *S. aureus* but had a weaker effect on *E. coli*. In contrast, the glycoside aucubin, isolated from *Aucuba japonica* leaves, exhibited no antimicrobial activity against either *S. aureus* or *E. coli*. These findings suggest that the antibacterial activity of the aglycone results from its ability to significantly inhibit RNA and protein synthesis in bacterial cells (Davini et al. 1986; Lee et al. 1986).

Similarly, other studies reported that the aglycone of galioside was active against *S. aureus* and *Klebsiella pneumoniae*, whereas the aglycone of gardenoside showed no such activity. This indicates that the antibacterial potential of these compounds depends on structural differences between their aglycones (Ishiguro et al. 1983).

Ajugol (**2**) and ajugoside (**3**) isolated from the aerial parts of *Sideritis lycia* Boiss. and Heldr. The antimicrobial activity of these compounds was evaluated against *B. subtilis* ATCC 6633, *Staph. aureus* ATCC 25923, *E. coli* ATCC 25882, *P. aeruginosa* RSKK 356 and Fungi *Candida albicans* ATCC 64550, and *Cryptococcııs neofornıans* (Akcoş et al. 1998).

Furthermore, Plumieride (**4**), protoplumericin A (**5**), and plumieride acid (**6**), three iridoids isolated from the bark and leaves of *Plumeria alba* L. They displayed distinct activity against some pathogenic bacteria and fungi (Afifi et al. 2006).

In addition, more studies that reported the antibacterial activity of iridoids as follows: Gentiopicroside (**7**), a secoiridoid glycoside with significant activity against *Serratia marcescens* (Kumarasamy et al. 2003b). Additionally, moderate antibacterial activity against *B. cereus*,* B. pumulus*, *B. subtilis*, *Micrococcus kristinae*, *S. aureus*, *E. coli*, *Klebsiella pneumoniae*, *P. aeruginosa*, and *Enterobacter cloacae* was detected when sweroside (**8**) isolated from the rootstock of *Scabiosa columbria* (Dipsacaceae) was used (Horn et al. 2001).

According to Modaressi et al. (2009), all three iridoids, phloyoside I (9), phlomiol (10), and pulchelloside I (11) isolated from the rhizomes of *Eremostachys laciniata* (family Lamiaceae), demonstrated low to moderate antibacterial activity with MIC values between 0.05 and 0.50 mg/mL. Among these, pulchelloside I (11) demonstrated the highest activity, against nine of the twelve tested bacterial strains. The most pronounced effects of pulchelloside I (11) were observed against *B.cereus*, *penicillin-resistant Escherichia coli*, *Proteus mirabilis*, and *S. aureus*, each with an MIC value of 0.05 mg/mL. None of the three iridoids exhibited inhibitory activity against *Klebsiella aerogenes*, *Lactobacillus plantarum*, or *methicillin-resistant S. aureus (MRSA).* Due to their structural similarity, phloyoside I (9), phlomiol (10), presented similar antibacterial activity.

Moreover, the crude extract obtained from *Canthium multiflorum’s* aerial parts, an iridoid called 6-oxo-genipin (**12**), and twelve other recognized chemicals were isolated. The antibacterial properties of the isolated compounds were tested on five distinct bacterial strains: S. aureus (DSM 799), *Actinobacter calcoaceticus* (DSM 30006), *Serratia plymuthica* (DSM 4540), *Pseudomonas stutzeri* (DSM 4166), and *Escherichia coli* (DSM 1116). Significant inhibitory efficacy was shown by 6-oxo-genipin **(12)** against all bacterial strains, specifically *S. aureus* (Kouam et al. 2013).

Six iridoid derivatives were isolated from the ant-plant *Myrmecodia tuberosa*. Two compounds among the isolated iridoids, asperulosidic acid (**13**) and deacetylasperulosidic acid (**14**), have moderate antibacterial activity on *S. aureus* with a MIC value of 100.0 μg/mL (Hanh et al. 2016).

Plumericin (**15**), an iridoid lactone, was isolated with relatively high yield from *Momordica charantia* vine. This compound showed antibacterial activity against *Enterococcus faecalis* and *Bacillus subtilis* (Saengsai et al. 2015).

Moreover, in the study of Joubouhi et al. (2017a, b), Canthiumoside (**16**) and linearin (**17**) had pronounced antibacterial activity against *Staphylococcus aureus* (Joubouhi et al. 2017a).

Physaloside A (**18**) and swertiamarin **(19**) were isolated from the ethanol extract of *Physochlaina physaloides* (L.) G. Don. The antibacterial activities of compounds **18** and **19** was evaluated and compared with zerumbone. Compound **18** demonstrated statistically comparable growth inhibition against *Yersinia enterocolitica* and *Bacillus cereus*, and lower activity against *E. coli* and *S. aureus*., in comparison to zerumbone. Compound **19** showed similar activity as compound **18** against *B. cereus*. Both compounds produced a statistically comparable growth inhibition pattern against *B. cereus*, *E. coli*, and *Y. enterocolitica* at varying doses (Wang et al. 2018).

Two bis-iridoids, namely 7-*O*-caffeoyl-sylvestroside I (**20**) and 7-*O*-(p-coumaroyl)-sylvestroside I (**21**), in addition to ten known compounds, were extracted from *Scabiosa stellata*. The antibacterial activity of the isolated compounds was evaluated on a total of 17 bacterial species. Nine extracted compounds showed strong antibacterial activity; in particular, compounds **20** and **21** had MIC values of 31.2 μg/mL against *Enterococcus faecalis* and 62.5 μg/mL against *S. epidermidis* (Lehbili et al. 2018).

Furthermore, Specioside glycoside isolated from the stem bark of *Tabebuia aurea* exhibited no antibacterial activity against *S. aureus*, *S. epidermidis* ATCC 35984, or *P. aeruginosa*. Additionally, it could not inhibit biofilm formation or destroy the biofilm already formed by these bacterial strains (Nocchi et al. 2020).

In the study of Aničić and others (2021*us*), the two Nepeta species and their main iridoids, cis, trans-nepetalactone (**22**) and trans, cis-nepetalactone (**23**), as well as 1,5,9-epideoxyloganic acid (1,5,9-eDLA) (**24**), were compared for their antibacterial and immunomodulatory properties. The isolated iridoids (trans, cis-nepetalactone and cis, trans-nepetalactone) and the iridoid glucoside 1,5,9-epideoxyloganic acid exhibited antibacterial activity. Isolated iridoids displayed antibiofilm potential against *P. aeruginosa*. where trans, cis-nepetalactone was the most active antibiofilm agent. The isolated iridoids also showed significant antifungal activity higher than ketoconazole against all tested fungi (Aničić et al. 2021).

Iridoid glycosides isolated from *Rehmannia glutinosa*, a widely used traditional Chinese medicinal plant, exhibited significant antibacterial activity against *S. aureus*. The MIC was determined to be 10.4 mg/mL, and time–kill assays showed that iridoid glycosides at 1 × MIC suppressed *S. aureus* growth within 5 h, while 3 × MIC concentrations achieved 99.9% bacterial reduction within 1 h, confirming their potent bactericidal effect (Zhang and Xu, 2024).

## Comprehensive overview of antimicrobial mechanisms and specific actions of iridoids

**Fig. 3:**
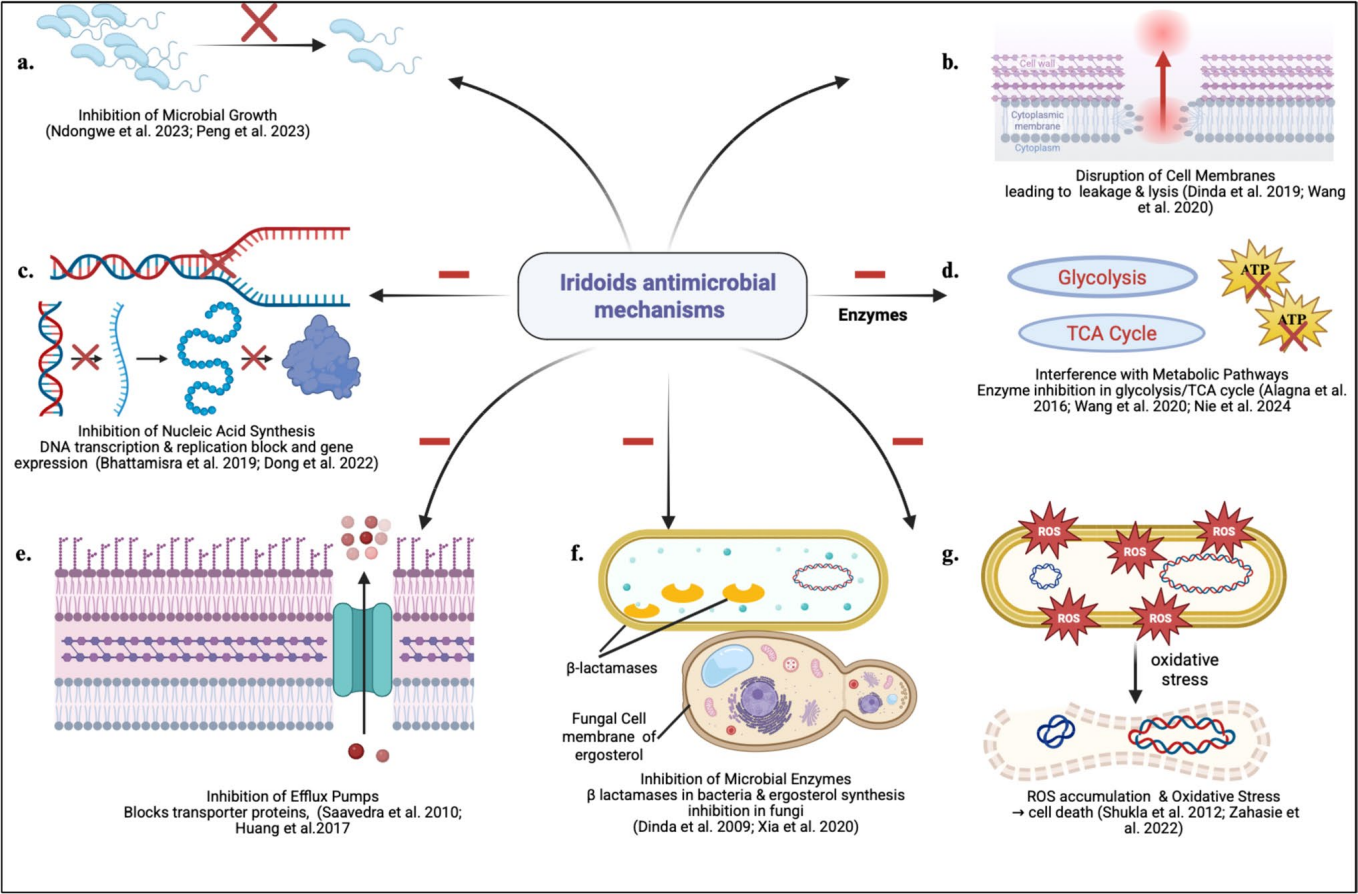
Common antimicrobial mechanisms of iridoids. ROS: reactive oxygen species; TCA cycle; tricarboxylic acid cycle

Iridoids have garnered significant attention for their broad-spectrum pharmacological activities, particularly in antimicrobial applications (Fig. 3). Their efficacy in inhibiting microbial growth positions them as promising candidates for treating and preventing infections, especially in light of escalating antibiotic resistance (Zhang and Xu 2024). A thorough understanding of the mechanisms underlying the antimicrobial actions of iridoids is crucial for optimizing their therapeutic potential and exploring their integration into existing treatment regimens (Wang et al. 2020; Arraché Gonçalves et al. 2022).

Iridoids are primarily distinguished by their complex bicyclic ring structures, which impart a range of biochemical activities that influence microbial cell integrity, metabolic pathways, and genetic material (Wang et al. 2024a). This structural versatility gives iridoids an advantage in targeting a variety of microorganisms, including bacteria, fungi, and even certain viruses. Their natural occurrence in various medicinal herbs makes them accessible, and their use as a complement or alternative to conventional antibiotics has become a growing area of research. Given their intricate molecular framework, iridoids present multiple possibilities for modifying microbial physiology, thereby offering novel opportunities for enhancing current therapeutic strategies (Qadri et al. 2022; Saini et al. 2024).

Iridoids exert their antimicrobial effects through various mechanisms that are context-dependent, varying with the type of microorganism, the specific iridoid compound, and environmental conditions (Przybylska et al. 2023). This section provides a comprehensive analysis of these mechanisms and the specific actions of iridoids, offering insights into their potential clinical applications as antimicrobial agents. However, detailed mechanistic studies remain limited, with most research focusing on activity profiles and structure–activity relationships. This suggests the need for more molecular and cellular studies, especially in clinically relevant and resistant pathogens, to clarify the precise mechanisms underlying their antimicrobial effects.

Herein, the antimicrobial mechanisms of iridoids are classified into several distinct categories:

### Inhibition of microbial growth

The antimicrobial activity of iridoids is primarily determined by their unique chemical structure, featuring a cyclopentane ring fused to a six-membered oxygen-containing ring, which facilitates specific interactions with microbial cells. These interactions initiate a cascade of biochemical disruptions that are lethal to pathogens (Ndongwe et al. 2023; Peng et al. 2023).

### Disruption of cell membrane integrity

Many antimicrobial agents exert their effects by targeting microbial cell membranes, resulting in altered permeability, leakage of intracellular components, and cell death (Zhang and Xu, 2024). Iridoids, such as geniposide and aucubin, possess amphiphilic and lipophilic properties that enable them to integrate into microbial membranes, compromising structural integrity and disrupting function (Dinda et al. 2019; Wang et al. 2020). This action is particularly effective in Gram-positive bacteria, which have simpler cell wall structures compared to Gram-negative bacteria, making them more vulnerable to such interventions. The incorporation of iridoids into lipid bilayers destabilizes the membrane, causing significant structural disturbances that lead to cell lysis and death. These compounds also interact with specific phospholipid components, enhancing their ability to disrupt membrane stability. The disruption extends to ion gradients and impairs membrane-bound enzyme functions, leading to loss of membrane potential and cellular homeostasis (Radulovic et al. 2013). Additionally, iridoids have been observed to aggregate at lipid rafts, specialized membrane microdomains critical for cell signaling and transport, further exacerbating membrane destabilization and impairing essential microbial communication processes. This multifaceted destabilization not only proves lethal to pathogens but also increases their susceptibility to co-administered antimicrobial agents (Guglielmi et al. 2020).

### Inhibition of nucleic acid synthesis

Certain iridoids, such as loganin and catalpol, can bind directly to microbial DNA, altering its helical structure and interfering with the enzymes essential for DNA replication and RNA transcription. This binding may involve intercalation, where the iridoid molecule inserts between base pairs, disrupting the DNA double helix and preventing the unwinding of DNA. These interactions inhibit microbial proliferation, especially in rapidly dividing bacterial populations, by halting genetic processes necessary for replication (Bhattamisra et al. 2019). Additionally, iridoids can obstruct RNA polymerase activity, preventing mRNA transcription. This dual impact, on both nucleic acid synthesis and gene expression, disrupts essential microbial functions, leading to growth inhibition and, ultimately, cell death (Wang et al. 2020). Furthermore, iridoids have been observed to influence the expression of genes involved in replication, suggesting their potential role in epigenetic modulation, offering a powerful means of suppressing pathogenic activity (Dong et al. 2022).

### Interference with metabolic pathways

Iridoids can disrupt microbial growth by inhibiting key enzymes involved in essential metabolic processes. This inhibition leads to a deprivation of critical metabolites or energy sources, ultimately hindering microbial proliferation (Wang et al. 2020). For instance, iridoids can act on enzymes involved in glycolysis or the tricarboxylic acid (TCA) cycle, thereby impairing the energy production necessary for cellular functions (Nie et al. 2024). Furthermore, the specificity of iridoid–enzyme binding often depends on the structural conformation of the iridoid molecule, allowing for targeted inhibition of critical metabolic pathways that are indispensable for microbial survival. Moreover, iridoids may exert an allosteric modulation effect, which changes the enzyme’s functional conformation, thereby impairing its activity even in the presence of substrate molecules. Such targeted disruption of metabolic processes not only inhibits microbial growth but also weakens pathogen virulence, making it more manageable by the host’s immune defenses (Alagna et al. 2016; Wang et al. 2020).

### Efflux pump inhibition and resistance modulation

Efflux pumps, particularly in multidrug-resistant bacterial strains, are critical in expelling antimicrobial agents from the cell, thereby diminishing their efficacy (Nanjan and Bose 2024). Iridoids have been shown to inhibit these efflux pumps, which increases the intracellular concentration of antimicrobial agents. This action not only sensitizes bacteria to the effects of iridoids but also suggests a potential role for iridoids as resistance-modulating agents in combination therapies. The inhibition of efflux pumps involves the binding of iridoids to the transporter proteins, thereby blocking the active transport of toxic compounds out of the cell. This mechanism is especially important for treating infections caused by multidrug-resistant bacteria, where efflux pump overexpression is a common resistance strategy. Notably, iridoids may also alter the expression of efflux pump-related genes, suggesting a capacity to downregulate these resistance mechanisms at the transcriptional level, thereby providing a multi-layered approach to overcoming resistance (Saavedra et al. 2010; Huang et al. 2017; Oyedemi et al. 2020).

### Enzymes’ inhibition

Iridoids can inhibit key enzymes crucial for microbial survival. For instance, iridoids have been demonstrated to inhibit β-lactamase, an enzyme that provides resistance to β-lactam antibiotics in certain bacteria. By inhibiting β-lactamase, iridoids can restore the efficacy of these antibiotics (Dinda et al. 2009). Additionally, in fungi, iridoids inhibit the synthesis of ergosterol, an essential sterol component of fungal cell membranes, thereby compromising cell membrane integrity and leading to cell death (Xia et al. 2020). The inhibition of these enzymes involves the formation of stable complexes between the iridoid and the enzyme’s active site, thereby preventing substrate binding and subsequent enzymatic activity. Such targeted enzyme inhibition highlights the specificity of iridoids in their antimicrobial action. Recent studies suggest that iridoids may also have a broader impact on enzymatic activity by modifying the enzyme’s allosteric sites, thereby indirectly affecting catalytic efficiency. This dual capacity for direct active site inhibition and allosteric modulation positions iridoids as versatile enzyme inhibitors capable of disrupting multiple biochemical pathways (Šiler et al. 2010; Aničić et al. 2021).

### Modulation ofreactive oxygen species (ROS) and oxidative stress

Certain iridoids, especially those with phenolic substituents, facilitate the accumulation of ROS within microbial cells. By disrupting the redox balance, these iridoids induce oxidative stress, which damages DNA, proteins, and lipids. The resultant accumulation of ROS overwhelms the antioxidant defenses, causing irreversible cellular damage and ultimately leading to cell death (Shukla et al. 2012). The pro-oxidant activity of iridoids is often accompanied by the inhibition of microbial antioxidant enzymes, such as catalase and superoxide dismutase, further exacerbating oxidative stress. This dual action of promoting ROS generation while inhibiting antioxidant defenses makes iridoids potent agents against a wide range of pathogens. Moreover, iridoids may modulate microbial redox-sensitive signaling pathways, further increasing oxidative stress by disrupting normal cellular signaling processes. The ability to target both the generation and neutralization of ROS underscores the comprehensive approach of iridoids in creating an inhospitable environment for microbial survival (Zaharie et al. 2022).

## Structure activity relationship (SAR) of iridoids

The structure–activity relationship of plant iridoids highlights the essential influence of their chemical configurations on biological functions and health advantages (Saidi et al. 2020). The cyclopentanopyran ring system, along with functional group substitutions and glycosylation patterns, plays a crucial role in determining their antioxidant, anti-inflammatory, and antimicrobial activities. Lipophilic substitutions, including methyl or acyl groups, have the potential to enhance membrane permeability, thereby improving the bioaccessibility of aglycones in lipid environments (Wang et al. 2024a). Furthermore, differences in the placement and nature of substituents, including carboxyl or methoxy groups, can affect binding affinities to biological targets, thereby impacting anti-inflammatory and antimicrobial effectiveness (Hernández Lozada et al. 2022). For instance, iridoid glycosides generally exhibit enhanced bioactivity upon hydrolysis to their aglycone forms, which increases their interaction with microbial targets. Several studies highlight that substitution patterns at key positions, such as hydroxyl groups at C-5 and methyl groups near carboxyl sites, play pivotal roles in modulating antimicrobial effects. The stereochemistry of iridoids, including the configuration of diastereoisomers like *cis*,*trans*- and *tran*s,*cis*-nepetalactones, also contributes to differential bioactivity profiles (Ishiguro et al. 1983; Aničić et al. 2021).

Topical anti-inflammatory activity is increased by hydroxyl substitution at C-5, unsaturation at C7–C8, and methyl substitution of carboxyl C-11 (del Carmen Recio et al. 1994). The C-7 hydroxyl group was replaced by a long-chain lipid ether to increase anticancer action. The substitution with short-chain fat ether decreased anticancer efficacy but increased osteoblast growth. C-11 is mostly a stable ester. Anti-cell adhesion and anti-diabetes efficacy increased with C-11 ester hydrolysis to acid or condensation to amide. The most efficient anti-diabetes structure is aromatic amide with halogen. Indole group-containing amides reduce cancer efficacy. Aniline or unsaturated ketene replaced C-11 to boost insulin production, with p-methoxy-aniline derivatives being the most active (Wang et al. 2024b). The insights gained from SAR are essential for comprehending the functional properties of iridoids and enhancing their application in functional foods and nutraceuticals.

## Future perspectives

Novel therapeutic alternatives are critical for combating antimicrobial resistance bacteria and lowering the burden of infectious illnesses. Bioactive compounds from medicinal plants, alone or in combination with known antimicrobials, offer viable solutions for addressing global antimicrobial resistance challenges. Plant-based antibiotic alternatives are appealing because they are less expensive, more widely available, safer, have less side effects, lower antimicrobial resistance risks, and help alleviate undesirable antibiotic effects and symptoms. Natural compounds, i.e., monoterpenoids, such as iridoids, are the main phytoconstituents accountable for the treatment of antimicrobial resistance such as recurrent urinary tract infections. Until now, current research has merely identified them, and knowledge on their modes of action is limited. combination therapies are powerful protocols to cure the infection caused by multidrug resistant strains. Moreover, the combination of iridoids with other phytochemicals such as phenolic acids may result in additive or synergistic antimicrobial interactions, further enhancing efficacy. In the future, the synergistic action of nanoparticles and iridoids may be used as an alternative therapy to combat bacterial resistance, with applications in particular industries (e.g., cosmetics, food, and pharmaceuticals) and healthcare facilities. Furthermore, nanoparticles can deliver antibiotics to affected cells while reducing drug dose and toxicity. Before employing nanoparticle-based treatment on humans, the suitable and effective dosage must be thoroughly investigated, and additional clinical studies are required. In vivo characterization of iridoids can reveal their pharmacodynamic and therapeutic potential.

## Conclusion

These diverse mechanisms suggest significant potential for iridoids as broad-spectrum antimicrobial agents, particularly in combination therapies aimed at enhancing the efficacy of conventional antibiotics and antifungal agents. Continued research into the structural optimization of iridoids and their synergistic potential with other antimicrobial agents could pave the way for new, effective treatment options in the fight against resistant infections. Formulation challenges include poor solubility and stability, which hinder effective delivery and sustained biological activity. Advances in drug delivery systems, such as encapsulation and nanoformulations, are being explored to overcome these barriers. Comprehensive toxicological evaluations are necessary to determine safe dosing ranges for therapeutic applications. Furthermore, understanding the underlying molecular mechanisms of iridoid interactions could lead to the development of novel derivatives with enhanced activity and specific it

## Supplementary Information

Below is the link to the electronic supplementary material.

ESM 1 (DOCX 1.03 MB)

## Data Availability

All source data for this work (or generated in this study) are available upon reasonable request.
