# Supplementary material for: Antimicrobial and antibiofilm actions of iridoids: a comprehensive mechanistic review
Source: Naunyn Schmiedebergs Arch Pharmacol. 2026 Mar 2;399(8):11117–32. doi: 10.1007/s00210-026-05063-9 (PMC13269524; doi:10.1007/s00210-026-05063-9)
Supplement: Supplementary file 1 — (DOCX 1.03 MB) [file 210_2026_5063_MOESM1_ESM.docx]

**Table S1**. Examples of iridoids isolated from natural sources.

|  | Name | Structure | Plant sources | Reference |
| --- | --- | --- | --- | --- |
| *Iridoid Glycosides* | | | | |
| 1 | Agnuside |  | *Vitex agnus-castus* (Lamiaceae) | *(Pandey et al., 2012)* |
| 2 | Ajugol |  | *Ajuga reptans*  (Lamiaceae) | *(Yi et al., 2022)* |
| 3 | Asperuloside |  | *Asperula odorata*  (Rubiaceae) | *(He et al., 2018; Rong et al., 2020)* |
| 4 | Asperulosidic acid |  | *Asperula odorata*  (Rubiaceae) | *(He et al., 2018; Rong et al., 2020)* |
| 5 | Aucubin |  | *Plantago asiatica*  (Plantaginaceae) | *(Carrillo-Ocampo et al., 2013; Bridi et al., 2023; Rahamouz-Haghighi, 2023)* |
| 6 | Barlerin |  | *Barleria dinteri*  (Acanthaceae) | *(Gololo et al., 2017)* |
| 7 | Boschnaloside |  | *Boschnakia rossica*  (Orobanchaceae) | *(Lan et al., 2018)* |
| 8 | Catalpol |  | *Rehmannia glutinosa*  (Orobanchaceae) | *(Carrillo-Ocampo et al., 2013; Bridi et al., 2023; Rahamouz-Haghighi, 2023)* |
| 10 | Geniposide |  | *Gardenia jasminoides*  (Rubiaceae) | *(Liu et al., 2022)* |
| 11 | Geniposidic acid |  | *Eucommia ulmoides*  (Eucommiaceae) | *(Cheng et al., 2022)* |
| 12 | Harpagoside |  | *Harpagophytum procumbens*  (Pedaliaceae) | *(Georgiev et al., 2013)* |
| 13 | Lamiide |  | *Lamium album*  (Lamiaceae) | *(Delaporte et al., 2002)* |
| 14 | Loganin |  | *Cornus officinalis*  (Cornaceae) | (Xu et al., 2022) |
| 15 | Loganic acid |  | *Cornus officinalis*  (Cornaceae) | (Xu et al., 2022) |
| 16 | Lupulinoside |  | *Barleria lupulina*  (Acanthaceae) | *(Suksamrarn et al., 2003)* |
| 17 | Morroniside |  | *Cornus officinalis*  (Cornaceae) | *(Bhuia et al., 2024)* |
| 18 | Mussaenoside |  | *Kickxia ramosissima*  (Scrophulariaceae) | *(Amin et al., 2017)* |
| 20 | Piasezkiiosides A |  | *Rehmannia piasezkii*  (Orobanchaceae) | *(Zhou et al., 2024)* |
| 21 | Piasezkiiosides B |  | *Rehmannia piasezkii*  (Orobanchaceae) | *(Zhou et al., 2024)* |
| 22 | Picroside II |  | *Picrorhiza kurroa*  (Plantaginaceae) | *(Li et al., 2020)* |
| 23 | Plantarenaloside |  | *Plantago sempervirens*  (Plantaginaceae) | *(Venditti et al., 2012)* |
| 24 | Prionitoside A |  | *Barleria prionitis*  (Acanthaceae) | *(Keomanykham et al., 2024)* |
| 25 | Prionitoside B |  | *Barleria prionitis*  (Acanthaceae) | *(Keomanykham et al., 2024)* |
| 26 | Rehmaglutoside A |  | *Rehmannia glutinosa*  (Orobanchaceae) | *(Liu et al., 2012)* |
| 27 | Rehmaglutoside B |  | *Rehmannia glutinosa*  (Orobanchaceae) | *(Liu et al., 2012)* |
| 28 | Rehmaglutoside C |  | *Rehmannia glutinosa*  (Orobanchaceae) | *(Liu et al., 2012)* |
| 29 | Rehmaglutoside D |  | *Rehmannia glutinosa*  (Orobanchaceae) | *(Liu et al., 2012)* |
| 30 | Rehmaglutoside E |  | *Rehmannia glutinosa*  (Orobanchaceae) | *(Liu et al., 2012)* |
| 31 | Rehmaglutoside F |  | *Rehmannia glutinosa*  (Orobanchaceae) | *(Liu et al., 2012)* |
| 32 | Rehmaglutoside G |  | *Rehmannia glutinosa*  (Orobanchaceae) | *(Liu et al., 2012)* |
| 33 | Rehmaglutoside H |  | *Rehmannia glutinosa*  (Orobanchaceae) | *(Liu et al., 2012)* |
| 34 | Rehmaglutoside I |  | *Rehmannia glutinosa*  (Orobanchaceae) | *(Liu et al., 2012)* |
| 35 | Rehmaglutoside J |  | *Rehmannia glutinosa*  (Orobanchaceae) | *(Liu et al., 2012)* |
| 36 | Rehmaglutoside K |  | *Rehmannia glutinosa*  (Orobanchaceae) | *(Liu et al., 2012)* |
| 37 | Rehmaglutoside L |  | *Rehmannia glutinosa*  (Orobanchaceae) | *(Liu et al., 2024b)* |
| 38 | Rehmaglutoside M |  | *Rehmannia glutinosa*  (Orobanchaceae) | *(Liu et al., 2024b)* |
| 39 | Rehmaglutoside N |  | *Rehmannia glutinosa*  (Orobanchaceae) | *(Liu et al., 2024b)* |
| 40 | Rehmaglutoside O |  | *Rehmannia glutinosa*  (Orobanchaceae) | *(Liu et al., 2024b)* |
| 41 | Sidichotomoside |  | *Sideritis dichotoma*  (Lamiaceae) | *(Öztürk et al., 2024)* |
| *Secoiridoid Glycosides* | | | | |
| 1 | Amarogentin |  | *Gentiana lutea*  (Gentianaceae) | *(Niu et al., 2016)* |
| 2 | Gentiopicroside |  | *Gentiana lutea*  (Gentianaceae) | *(Wang et al., 2023)* |
| 3 | Ligustroside |  | *Ligustrum lucidum*  (Oleaceae) | *(DiAz et al., 2000)* |
| 4 | Oleuropein |  | *Olea europaea*  (Oleaceae) | *(Omar, 2010)* |
| 5 | Maytenhoiridosides A |  | *Maytenus hookeri*  (Celastraceae) | *(Lv et al., 2024)* |
| 6 | Maytenhoiridosides B |  | *Maytenus hookeri*  (Celastraceae) | *(Lv et al., 2024)* |
| 7 | Swertiamarin |  | *Swertia chirayita*  (Gentianaceae) | *(Fadzil et al., 2021)* |
| *Non-Glycosidic Iridoids* | | | | |
| 1 | Acevaltrate |  | *Valeriana glechomifolia*  (Valerianaceae) | *(Mi et al., 2024)* |
| 2 | Aucubigenin |  | *Pseudolysimachion linariifolium*  (Plantaginaceae) | *(Xue et al., 2022)* |
| 3 | Aureanin |  | *Tabebuia aurea*  (Bignoniaceae) | *(Mahmoud et al., 2024)* |
| 4 | Catalpin A |  | *Catalpa ovata*  (Bignoniaceae) | *(Zhang et al., 2023)* |
| 5 | Catalpin B |  | *Catalpa ovata*  (Bignoniaceae) | *(Zhang et al., 2023)* |
| 6 | Dihydrovaltrate |  | *Valeriana officinalis*  (Valerianaceae) | *(Andreatini et al., 2002)* |
| 7 | Eucommiate A |  | *Eucommia ulmoides*  (Eucommiaceae) | *(Lv et al., 2023)* |
| 8 | Eucommiol-2-hydroxypropylate |  | *Eucommia ulmoides*  (Eucommiaceae) | *(Lv et al., 2023)* |
| 9 | Garjasmine |  | *Rothmannia wittii*  (Rubiaceae) | *(Chaipukdee et al., 2016)* |
| 10 | Jiofuraldehyde |  | *Catalpa ovata*  (Bignoniaceae) | *(Zhang et al., 2023)* |
| 11 | Ovatofuran A |  | *Catalpa ovata*  (Bignoniaceae) | *(Zhang et al., 2023)* |
| 12 | Ovatofuran B |  | *Catalpa ovata*  (Bignoniaceae) | *(Zhang et al., 2023)* |
| 13 | Ovatofuran C |  | *Catalpa ovata*  (Bignoniaceae) | *(Zhang et al., 2023)* |
| 14 | Patrinoside G |  | *Patrinia scabiosaefolia*  (Valerianaceae) | *(Liu et al., 2023)* |
| 15 | Patrinoside H |  | *Patrinia scabiosaefolia*  (Valerianaceae) | *(Liu et al., 2023)* |
| 16 | Rupesin F |  | *Valeriana jatamansi*  (Valerianaceae) | *(Maurya and Agnihotri, 2024)* |
| 17 | Scabrol B |  | *Patrinia scabra*  (Valerianaceae) | *(Li et al., 2024)* |
| 18 | Scabrol C |  | *Patrinia scabra*  (Valerianaceae) | *(Li et al., 2024)* |
| 19 | Teucriifolian A |  | *Nepeta teucriifolia*  (Lamiaceae) | *(Eser et al., 2023)* |
| 20 | Teucriifolian B |  | *Nepeta teucriifolia*  (Lamiaceae) | *(Eser et al., 2023)* |
| 21 | Teucriifolian C |  | *Nepeta teucriifolia*  (Lamiaceae) | *(Eser et al., 2023)* |
| 22 | Valepotriates |  | *Valeriana officinalis*  (Valerianaceae) | *(Andreatini et al., 2002)* |
| 23 | Valeralide A |  | *Valeriana officinalis*  (Valerianaceae) | *(Liu et al., 2024a)* |
| 24 | Valeralide B |  | *Valeriana officinalis*  (Valerianaceae) | *(Liu et al., 2024a)* |
| 25 | Valeralide C |  | *Valeriana officinalis*  (Valerianaceae) | *(Liu et al., 2024a)* |
| 26 | Valeralide D |  | *Valeriana officinalis*  (Valerianaceae) | *(Liu et al., 2024a)* |
| 27 | Valeralide E |  | *Valeriana officinalis*  (Valerianaceae) | *(Liu et al., 2024a)* |
| *Bis Iridoids* | | | | |
| 1 | Cantleyoside |  | *Pterocephalus perennis*  (Dipsacaceae) | *(Graikou et al., 2002)* |
| 2 | Laciniatoside I |  | *Pterocephalus hookeri*  (Dipsacaceae) | *(Chen et al., 2018)* |
| 3 | Laciniatoside II |  | *Pterocephalus hookeri*  (Dipsacaceae) | *(Chen et al., 2018)* |
| 4 | Sylvestroside I |  | *Acicarpha tribuloides*  (Calyceraceae) | *(Capasso et al., 1996)* |
| 5 | Sylvestroside III |  | *Acicarpha tribuloides*  (Calyceraceae) | *(Capasso et al., 1996)* |
